# Short-term clinical outcomes in single-stage vs. two-stage corrective surgery after pedicle subtraction osteotomy for degenerative deformities in an elderly cohort

**DOI:** 10.1016/j.bas.2026.106128

**Published:** 2026-06-17

**Authors:** Claudius Jelgersma, Daria Samoylenko, Anton Früh, Kiarash Ferdowssian, Robert Mertens, Christian Entenmann, Ahmad Almahozi, Dimitri Tkatschenko, Tarik Alp Sargut, Nils Hecht, Peter Vajkoczy, Lars Wessels

**Affiliations:** Department of Neurosurgery, Charité – Universitätsmedizin Berlin, Germany

**Keywords:** Staged surgery, Elderly, Spinal deformity, Corrective spine surgery, Complications, Pedicle subtraction osteotomy

## Abstract

**Objective:**

Corrective spinal surgery involving pedicle subtraction osteotomy is a demanding procedure associated with a high risk of complications, particularly in elderly patients. Staged approaches have been proposed to reduce perioperative risk and patients' burden; nevertheless, evidence in this population is limited.

**Research question:**

This study compares single versus two-staged approaches in elderly patients, assessing short-term outcomes and complication severity.

**Methods:**

Fifty-one patients who underwent corrective spinal fusion with pedicle subtraction osteotomy between 2014 and 2023 were retrospectively analyzed. Clinical characteristics and complications were compared, with complications classified using the modified Clavien–Dindo–Sink classification.

**Results:**

The median age was 70 and 71 years in the single-stage group (1-SG, n = 30) vs. the two-stage group (2-SG, n = 21). Baseline characteristics and the number of instrumented segments (1-SG: 9.7 ± 4.2; 2-SG: 10.6 ± 3.4) were comparable. Cumulative operative time was significantly shorter in 1-SG (431 ± 131 min) compared with 2-SG (653 ± 142 min). However, perioperative parameters, including estimated blood loss, transfusion rate, hospital stay, and discharge modality, showed no significant differences. The incidence and severity of complications within 90 days were also comparable across both groups.

**Conclusions:**

This study demonstrates no beneficial effect of staged surgery for corrective spine surgery in elderly patients. The findings suggest that overall surgical invasiveness, rather than timing strategy, is more critical when prolonged operating times and high blood loss are expected.

## Highlights

• Single- and two-stage corrective spine surgery show similar complication rates in elderly.
• Staged surgery prolongs total operating time without improving short-term outcomes.
• Overall surgical invasiveness seems to outweighs timing strategy in elderly spine deformity surgery.

## 1 Introduction

Corrective spine surgery for adult degenerative deformity demonstrates positive clinical outcomes, even in the elderly population (Bridwell et al., 2009; Smith et al., 2011; Sciubba et al., 2016). However, a major challenge remains the high complication rate associated with these technically demanding and long hour-lasting operations (Smith et al., 2011; Alvarez et al., 2022). Overall, in spinal deformity surgery, clinical complication rates reach up to 21% and implant-associated complications in 16% can be assumed as reported in a meta-analysis (Alvarez et al., 2022). In smaller studies, the complication rate for open approaches for deformity correction was found to be as high as 45% (Haque et al., 2014) and when pedicle subtraction osteotomy (PSO) is performed, reported complication rates can reach up to 59.5% and even 78% (Smith et al., 2017; Corbett et al., 2024). Additionally, there is a considerable thirty-day mortality rate of 1.2% associated with these procedures (Carbone et al., 2025), which is of particular relevance for the aging population, as age is a known risk factor for frailty, and frailty itself increases the risk of complications and mortality (Alvarez et al., 2022; Miller et al., 2017; Chiu et al., 2024). Besides approaches aiming to improve patients’ outcome through better patient selection based on risk and frailty scores (Zileli and Dursun, 2020; Passias et al., 2025), strategies have been tested to divide multi-hour surgeries into staged procedures to reduce the burden on both patients and healthcare providers, including soft factors such as surgeon fatigue. However, the role of staged approaches remains an ongoing debate as current literature provides insufficient evidence on the elderly for an appropriate decision-making process, particularly in cases in which pedicle subtraction osteotomy is required to achieve adequate sagittal correction. Therefore, the present study compares single-stage versus two-stage surgical approaches in an elderly patient cohort undergoing corrective surgery in which PSO is required. The surgical outcomes were assessed based on operative time, estimated blood loss, transfusion rate, and discharge modality. Furthermore, the severity of complications was systematically classified and compared using the modified Clavien-Dindo-Sink classification for the first 90 days postoperatively.

## 2 Methods

Patients who underwent degenerative deformity surgery between 2014 and July 2023 were retrospectively screened for inclusion in the study conducted at our neurosurgical department. The inclusion criteria were adult patients with degenerative spinal deformity requiring corrective surgery involving a PSO. Both type III (pedicle + partial body) or type IV (pedicle + partial body + disc) osteotomy according to Schwab's classification (Schwab et al., 2015) were included, and surgery was performed in either a single- or two-stage surgical approach. The surgical strategy was determined according to the surgeon's preference, with consensus after case discussion among the most experienced spine surgeons performing these corrective spine surgeries. Within the case discussion, the patient's clinical status and the anticipated complexity of the required correction were taken into account, and decisions were based on clinical judgment rather than predefined thresholds for comorbidities or age. In the two-stage approach, PSO was performed during the second operation, whereas the first stage primarily involved soft-tissue preparation and pedicle screw placement. Patients undergoing the two-stage approach received standard postoperative care between surgeries, including pain management and physiotherapy. To ensure comparability between these approaches, only patients who underwent corrective surgery (via PSO in combination with a variable number of minor spinal osteotomies and interbody fusion cage implantations) entirely posteriorly were included. Hence, only patients without additional anterior/lateral/anterolateral approaches were included. Of the 121 patients reviewed, 51 patients were included (single-stage group (1-SG): n = 30; two-stage group (2-SG): n = 21). Clinical data were collected retrospectively from medical reports and record sheets. Complications documented within the first 90 days postoperatively were classified according to the modified Clavien-Dindo-Sink classification (Ridolfi et al., 2024). This classification was adapted to incorporate both general clinical and spine-specific complications. It is based on the original Dindo classification for surgical complications established in 2004 (Dindo et al., 2004). In summary: Grade I includes complications without deviation from the normal clinical course (e.g., electrolyte imbalances, temporary disorientation, uncomplicated urinary tract infections, transient extremity weakness); Grade II comprises complications deviating from the normal postoperative course that require non-invasive or minor interventions (e.g., mild paresis, blood transfusion, antibiotic therapy, thrombotic events such as deep vein thrombosis or pulmonary embolism); Grade III includes complications requiring invasive treatment (e.g., reoperation for postoperative bleeding, wound infection, implant displacement); Grade IV represents life-threatening complications and severe neurological deficits (IVa: transient neurological paralysis or paresis, organ failure, or sepsis; IVb: permanent neurological deficit such as spinal cord injury or irreversible nerve injury, severe organ dysfunction, or multi-organ failure); and Grade V denotes death (Ridolfi et al., 2024).

For statistical analyses and graphical design, IBM® SPSS® Statistics (Version 31.0.0.0) and GraphPad Prism (Version 10.2.3) software were used. Standard statistical tests were applied to compare patient characteristics and outcome parameters and are described in each section. For the comparison of complication rates, each complication was treated as an individual observation, resulting in multiple observations per patient. A generalized linear mixed-effects model with patient identity included as a random effect was used to appropriately account for within-patient clustering of complications. The surgical approach was set as a fixed effect. A significance level of p < 0.05 was assumed and adjusted using Bonferroni correction for multiple testing, resulting in an adjusted significance level of p < 0.0083.

## 3 Results

### 3.1 Baseline characteristics

Baseline characteristics are presented in detail in Table 1. None of the baseline parameters demonstrated a statistically significant difference between the two groups. The median age for both groups was 70 years (IQR: 64–76 years) in the 1-SG and 71 years (IQR: 63–75 years) in the 2-SG. The body mass index (BMI) had a median of 26 kg/m^2^ (IQR: 23–30 kg/m^2^) in the 1-SG and was slightly higher in the 2-SG with 30 kg/m^2^ (IQR: 25–35 kg/m^2^). The proportion of female patients was 53% in the 1-SG and 67% in the 2-SG. No ASA grade I patients were found in the 1-SG. The following ASA grading was recorded: 1-SG: grade II = 27%, grade III = 67%, and 6% were not available. In the 2-SG, 5% of patients were classified as ASA grade I, 48% as grade II, and 48% as grade III. In addition to ASA classification, smoking status and common comorbidities (as listed in Table 1) were assessed, with no significant intergroup differences observed. The main proportion of the patients in both groups underwent prior spinal surgery (87% in 1-SG and 90% in 2-SG). Baseline analysis revealed no significant differences between the groups regarding the radiographic severity of deformity, as assessed by spinopelvic parameters. The GAP score was likewise comparable between groups (Yilgor et al., 2017). The total number of instrumented segments was nearly equal (single-stage: mean 9.7 ± 4.2; two-stage: 10.6 ± 3.4 segments). The distribution of the upper instrumented vertebrae (UIV) was more variable in the 1-SG, with the highest proportion located at T10 (33%) or above (T1-T9, 33%). In the 2-SG, 57% of the patients were instrumented from T10 and only 24% from levels above (T1-T9). Only 29% in the 2-SG and 10% in the 1-SG received proximal tethering (UIV+2) (Sargut et al., 2025) (Translace™, Medtronic Sofamor Danek, Menphis, TN, USA). A higher proportion of patients in the 2-SG was instrumented with S2-ala-iliac screws (S2AI) (95% vs. 77% in the 1-SG). Interbody fusion techniques as well as Smith-Petersen osteotomies (SPOs) were performed more frequently in the 2-SG (posterior cage placement: 20% in 1-SG vs. 76% in 2-SG; at least one SPO: 10% in 1-SG vs. 38% in 2-SG; details in Table 1). The segment chosen for PSO was most commonly L3 (30%) or L4 (60%) in the 1-SG and L4 (81%) in the 2-SG.

**Table 1:** Patient characteristics – single-vs. two-stage.

		1-SG (n = 30)	2-SG (n = 21)	p
Age (years), median (IQR)		70 (64-76)	71 (63-75)	0,975

BMI (kg/m2), median (IQR)		26 (23-30)	30 (25-35)	0,053

Sex, female, n (%)		16 (53)	14 (67)	0,397

ASA score, n (%)	**I**		1 (5)	0,135
	**II**	8 (27)	10 (48)	
	**III**	20 (67)	10 (48)	
	**n.a.**	2(6)		

Smoking status active, n (%)		7 (23)	6 (29)	0.750

T2DM, n (%)		9 (30)	9 (43)	0.344
aHT, n (%)		16 (53)	14 (67)	0.341
HF, n (%)		2 (7)	5 (24)	0.109
CAD, n (%)		4 (13)	5 (24)	0.460
COPD, n (%)		2 (7)	0 (0)	0.506
PAD, n (%)		3 (10)	2 (10)	1
Previous TIA/Stroke, n (%)		5 (17)	1 (5)	0.381

Prior spinal surgery, n (%)	**None**	4 (13)	2 (10)	0,295
	**Decompression**	2 (7)		
	**Fusion**	24 (80)	19 (90)	

Preop. Spinopelvic parameters, mean (SD) PI (°)		56(13)	57(12)	0,774

	**PT (°)**	26(10)	31(8)	0,086
	**SS (°)**	26(10)	27(11)	0,757
	**Total LL (°)**	28(14)	25(12)	0,364
	**LL L4-S1 (°)**	23(13)	27(14)	0,359
	**PI-LL (°)**	28(18)	32(13)	0,335
	**SVA in (mm)**	119(45)	134(55)	0,290
	**T4-T12 (°)**	32(17)	33(22)	0,790
	**GT (°)**	41(14)	49(14)	0,076
	**GAP Score**	8,3(2,7)	9(5,6)	0,546

UIV, n (%)	**T2**	3 (10)	2 (9,5)	0,179
	**T3**	3 (10)	2 (9,5)	
	**T4**	2 (2,7)	1 (4,8)	
	**T8**	2 (2,7)	1 (4,8)	
	**T9**		1 (4,8)	
	**T10**	10 (33,3)	12 (57,1)	
	**T12**		1 (4,8)	
	**L1**	8 (26,7)	1 (4,8)	
	**L2**	2 (6,7)		

LIV, n (%)	**L4**	1 (3,3)		0,138
	**S1**	6 (20)	1 (4,8)	
	**S2AI**	23 (76,7)	20 (95,2)	

Total segments, mean (SD)		9,7 (4,2)	10,6 (3,4)	0,434

Total dorsal cages, n (%)		6 (20)	16 (76,2)	0,589
	**1**	4 (13)	10 (48)	
	**2**	1 (3)	6 (29)	
	**4**	1 (3)		

Total SPOs*, n (%)		3 (10)	8 (38)	0,559
	**1**	2 (6,7)	4 (19)	
	**2**	1 (3,3)	3 (14,3)	
	**3**		1 (4,8)	

PSO level	**T11**	2 (6,7)		0,227
	**L3**	9 (30)	3 (14,3)	
	**L4**	18 (60)	17 (81)	
	**L5**	1 (3,3)	1 (4,8)	

Prox. Tethering (UIV+2) **, n (%)		3 (10)	6 (29)	0,136

IQR: interquartile range. BMI: body mass index. ASA: American Society of Anesthesiologists. T2DM: type 2 diabetes mellitus. aHT: arterial hypertension. HF: heart failure. CAD: coronary artery disease (included patients with angina pectoris, previous stenting, or coronary bypass). PAD: peripheral artery disease. PI: pelvic incidence. PT: pelvic tilt. SS: sacral slope. LL: lumbar lordosis. SVA: sagittal vertical axis. UIV: upper instrumented level. LIV: lower instrumented level. SPO: Smith-Petersen Osteotomy. *Counted if it was documented additional to the segment of interbody fusion with the aim to gain more sagittal correction. PSO: Pedicle Subtraction Osteotomy. **translaminar spinal tethering (Translace™, Medtronic). Column “p” shows p-values of statistical comparisons. Chi-Square test, Fisher's exact test, and Student's t-test were used.

### 3.2 Comparison of surgical outcome parameters and complications

**Fig. 1:**
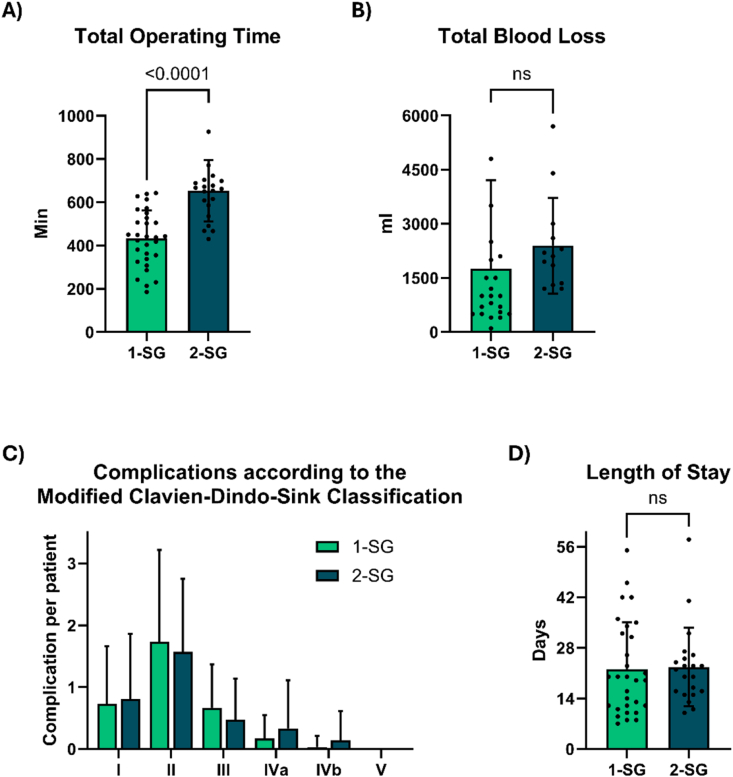
Outcome parameters single-vs. two-stage approach in the elderly (A) The 1-SG had a significantly shorter total operating time compared to the 2-SG (p < 0.001; Student's t-test), whereas (B) estimated blood loss did not differ significantly (n.s.; Student's t-test). (C) Complications were compared between groups using the modified Clavien-Dindo-Sink classification and are presented as the average number per grade per patient; no statistical difference was found (n.s.; generalized linear mixed-effects model). (D) The length of hospital stay in days was nearly identical between groups.

The total operating time in 1-SG was significantly lower (Student's t-test; p=<0.001) with a mean of 432 ± 131 min (median 440 min, IQR 335–534) compared to 653 ± 142 min (median 662 min, IQR 561–702; Fig. 1A). When analyzing the two surgical stages in 2-SG separately, mean operating times were 327 ± 101 min (median 325 min, IQR 274–381) for the first surgery and 326 ± 84 min (median 328 min, IQR 285–382) for the second. The mean time interval between the two surgeries was 6 ± 4.2 days (median: 5 days, IQR 3–7).

The total estimated blood loss in 1-SG was 1761 ± 2448 ml in mean (median 1000 ml, IQR 500–2025, n = 22), and was slightly higher in 2-SG without statistical significance (Student's t-test; p = 0.396), at 2396 ± 1327 ml (median 2100 ml, IQR 1325–2800, n = 13; Fig. 1B). When analyzing the first and second surgery in the 2-SG separately, a blood loss of mean 970 ± 605 ml in the first, and 1450 ± 886 ml in the second surgery was observed. The proportions of indicated transfusion of blood products did not differ (1-SG: 83% vs. 91%, p = 0.685). Both groups exhibited a comparable drop in haemoglobin levels following the first surgery (1-SG decreased from a mean 12.3 ± 2.1 g/dL to 8.8 ± 1.5 g/dL, and 2-SG from 13.2 ± 1.5 g/dL to 8.8 ± 1.3 g/dL postoperatively). In the 2-SG, haemoglobin increased to 9.2 ± 1.2 g/dL prior to the second surgery following transfusion and subsequently decreased to 8.5 ± 1.1 g/dL postoperatively. Differentiated analysis of individual complications and their severity according to the modified Clavien-Dindo-Sink classification within the first 90 days post surgery revealed no significant difference (generalized mixed-effect model; p = 0.833). For a comparative illustrated overview, complications per group and grade were averaged per patient (Fig. 1C). In addition, also binary comparison of complications requiring invasive treatment (grade ≥ III) and those managed non-invasively (grade < III) showed no group difference (generalized mixed-effect model; p = 0.711).

The mean cumulative length of stay in the intensive care unit was almost identical in both groups (1-SG: 4.7 ± 6.2 days vs. 2-SG: 4.3 ± 5.4 days). Similarly, the mean total length of hospital stay showed no significant differences (1-SG: 22.1 ± 13 days vs. 2-SG: 22.7 ± 10.8 days, p = 0.86; Fig. 1D). In the 1-SG, 33% of patients were discharged home, 17% to direct rehabilitation, and 50% to further inpatient care, whereas in the 2-SG, 38% were discharged home and 62% to ongoing inpatient care (Fisher's exact test; p = 0.195). Readmissions due to wound healing disorders requiring surgical revision within 90 days postoperatively were comparable, with proportions of 26.7% in the 1-SG and 28.6% in the 2-SG.

## 4 Discussion

This retrospective study examined short-term clinical outcomes of single-stage versus two-stage approaches in corrective spinal surgery in an elderly patient cohort requiring PSO for sagittal alignment correction. The findings indicate that, aside from the longer cumulative operative time in the two-stage group, key outcome parameters including blood loss, length of stay, discharge modality, and complications within the first 90 days post-surgery did not differ significantly.

Recent studies comparing single-stage and multi-staged spinal surgeries often involve inhomogeneous patient populations, which may introduce a selection bias (Passias et al., 2025; Daher et al., 2024; Yamato et al., 2021). Here, multi-staged surgeries typically encompass posterior instrumentation and anterior, lateral, or anterolateral interbody fusion techniques. To minimize this selection bias, we focused exclusively on adult degenerative deformity cases of patients who underwent posterior-only instrumentation in single- or two-stage approaches. Given that baseline characteristics did not reveal significant differences between groups, we consider them to be comparably aligned.

Although comparability with other published studies is limited by heterogeneity in surgical strategies, varying indications, and often substantially younger patient cohorts (mean age 49 years), one of the largest series, a meta-analysis of 16 retrospective studies including 2346 patients (Daher et al., 2024) similarly found no advantage for staged procedures. More specifically, Daher et al. concluded that in younger patients, single-stage strategies may be preferable, as no significant differences were observed in blood loss, mortality, revision rates, or non-home discharges, whereas longer operative times, hospital stays, and higher complication rates were associated with staged surgery.

As age is an independent risk factor for frailty, which correlates with higher complication rates and mortality, it is essential to evaluate elderly patients separately (Alvarez et al., 2022; Miller et al., 2017). In comparison to our study, only a few articles can be considered, although their limitations are compounded by the heterogeneity mentioned earlier. (Yamato et al., 2021; Arzeno et al., 2019). In these studies, patient average ages of 68 and 70 years closely align with our cohort. Notably, both studies by Yamato et al. and Arzeno et al. found no significant differences in complication rates among elderly patients based on the surgical strategy, although radiological outcomes were superior with the staged approach (Yamato et al., 2021; Arzeno et al., 2019).

To comprehensively assess all complications, including non-surgical ones, the modified Clavien-Dindo-Sink classification was applied (Ridolfi et al., 2024). This classification was selected due to its strong inter- and intrarater reliability when analyzing retrospective cohorts and its ability to capture both clinical and spine-specific complications. From the authors’ perspective, it was therefore more suitable than other available classifications, such as the SAVES-V2 score, may prove more effective in prospectively assessed complications. Supporting this, studies have shown that the incidence of adverse events increases from 23% in retrospective analyses to 87% in prospective analyses when the SAVES-V2 score is applied (Street et al., 2012; Rampersaud et al., 2016). Accordingly, the implementation of this more sensitive scoring system would require a prospective study design.

Interestingly, our study revealed no significant difference in overall complication rates between single-stage and two-stage approaches, aligning with findings from previously mentioned elderly cohorts (Yamato et al., 2021; Arzeno et al., 2019). Consequently, it was not unexpected that there were no differences in other outcome parameters, including length of stay and readmission rates due to wound infection. One possible interpretation of the absence of significant differences between the groups is that the decisive factor may not be the surgical strategy itself, but rather the overall invasiveness of the procedure. This may also explain why both the length of hospital stay and the duration in the intensive care unit were nearly identical in our cohort.

Alternatively, it must be considered that the non-randomized selection of surgical approach in our cohort may confound the observation, as patients undergoing two-stage procedures often require more complex corrective steps that might have resulted in less favorable outcomes if performed in a single stage. Since the two-stage group showed only a tendency toward a higher number of additional transforaminal lumbar interbody fusions and SPOs but did not reach statistical significance, this interpretation remains speculative.

In general, the complexity of corrective surgery, particularly with pedicle subtraction osteotomy, is evident in the reported outcomes from the literature. High complication rates are noted, ranging from 59.5% to 78%, along with a mortality risk of 1.2% within the first postoperative 30 days (Smith et al., 2017; Corbett et al., 2024; Carbone et al., 2025). Closer examination of independent risk factors in elective spine surgery of various indications reveals that longer operative times and higher blood loss correlate with increased complication rates. Specifically, a threshold for drastically increased risk has been identified at 3 to 5 h of operative time, a duration that was exceeded in the mean in both groups of our study (Hersey et al., 2019; Monetta et al., 2024; Findlay et al., 2024).

Similarly, most patients exceeded a mean estimated blood loss of more than 1 L regardless of whether the surgical approach was staged, which has also been reported as an independent risk factor for increased postoperative infections (Pull ter et al., 2009). A recently published article comparing staged and same-day procedures supports the idea that staging alone does not significantly predict optimal outcomes (Passias et al., 2025). Instead, advantages appear to be refined to specific subgroups of patients. The study found that better outcomes are associated with a Charlson Comorbidity Index of ≤1. In contrast, patients with an Edmonton Frail Scale score of ≥7, 9 or more fused levels, and post-operative pelvic incidence-lumbar lordosis (PI-LL) mismatches of ≥15.3 are more likely to achieve optimal outcomes when undergoing staged approaches (Passias et al., 2025).

## 5 Limitations

The primary limitations of this study are attributed to the nature of its retrospective design. The moderate quality of documentation resulted in incomplete outcome data for specific patient parameters, as detailed in the results section. Specifically, long-term outcomes were not consistently available due to loss to follow-up; therefore, the analysis was limited to a 90-day period. Whether complication rates, including hardware-related complications, differ between groups in the long term remains unclear. Furthermore, pain scores, quality of life questionnaires, and the surgeons' decision-making processes were not recorded consistently throughout the study period, which prevented their inclusion in the analysis. The limited sample size, combined with the relatively low frequency of complications, may reduce statistical power and increase the risk of type II error, thereby limiting the strength of conclusions regarding differences between surgical strategies. Lastly, it is important to note that there may be a significant selection bias, as our data indicates no notable differences in age or ASA score between the two groups, although such differences could have been expected, since more vulnerable patients would likely have driven the decision toward a staged approach.

## 6 Conclusions

This study indicated no significant difference in short-term surgical outcomes between single-stage and two-stage approaches in corrective spine surgery for elderly patients. This finding is consistent with published cohorts of similar age groups, although direct comparisons are limited due to the various surgical strategies described. The overall invasiveness of the procedure may be more critical to patient outcomes than the specific surgical approach itself.

## Ethical approval and informed consent statements

The study adhered to the principles outlined in the Declaration of Helsinki and received ethical approval from the local ethics committee (EA2/072/25). Given the retrospective study design, the requirement for informed consent was waived by the ethics committee.

## Author contributions

Conceptualization: Claudius Jelgersma, Anton Früh, Lars Wessels; Methodology: Claudius Jelgersma, Anton Früh, Lars Wessels; Data curation: Claudius Jelgersma, Christian Entenmann, Ahmad Almahozi; Formal analysis and investigation: Claudius Jelgersma, Daria Samoylenko, Kiarash Ferdowssian, Robert Mertens; Writing - original draft preparation: Claudius Jelgersma; Writing - review and editing: All authors; Resources: Dimitri Tkatschenko, Tarik Alp Sargut, Nils Hecht, Peter Vajkoczy, Lars Wessels; Supervision: Nils Hecht, Peter Vajkoczy, Lars Wessels<a name = "Line_manuscript_48">

## Declaration of generative AI and AI-assisted technologies in the manuscript preparation process

During the preparation of this work, the authors used Grammarly (2026) in order to improve grammar and language clarity. After using this tool, the authors reviewed and edited the content as needed and take full responsibility for the content of the published article.

## Funding

The authors received no financial support for the research, authorship, and/or publication of this article.

## Declaration of competing interest

The authors declare that they have no known competing financial interests or personal relationships that could have appeared to influence the work reported in this paper.
